# Spatio-temporal variations of PM_2.5_ concentrations and the evaluation of emission reduction measures during two red air pollution alerts in Beijing

**DOI:** 10.1038/s41598-017-08895-x

**Published:** 2017-08-15

**Authors:** Nianliang Cheng, Dawei Zhang, Yunting Li, Xiaoming Xie, Ziyue Chen, Fan Meng, Bingbo Gao, Bin He

**Affiliations:** 1 College of Water Sciences, Beijing Normal University, Beijing, 100875 China; 2 Beijing Municipal Environmental Monitoring Center, Beijing, 100048 China; 3 Chinese Research Academy of Environmental Sciences, Beijing, 100012 China; 4 Department of Environmental Science and Engineering, Tsinghua University, Beijing, 100084 China; 5 State Key Laboratory of Remote Sensing Science, College of Global Change and Earth System Science, Beijing Normal University, 19 Xinjiekouwai Street, Haidian, Beijing 100875 P.R. China; 6 National Engineering Research Center for Information Technology in Agriculture, 11 Shuguang Huayuan Middle Road, Beijing, 100097 China

## Abstract

To effectively improve air quality during pollution episodes, Beijing released two red alerts in 2015. Here we examined spatio-temporal variations of PM_2.5_ concentrations during two alerts based on multiple data sources. Results suggested that PM_2.5_ concentrations varied significantly across Beijing. PM_2.5_ concentrations in southern parts of Beijing were higher than those in northern areas during both alerts. In addition to unfavorable meteorological conditions, coal combustion, especially incomplete coal combustion contributed significantly to the high PM_2.5_ concentrations. Through the CAMx model, we evaluated the effects of emission-reduction measures on PM_2.5_ concentrations. Through simulation, emergency measures cut down 10% – 30% of the total emissions and decreased the peaks of PM_2.5_ concentrations by about 10–20% during two alerts. We further examined the scenario if emergency measures were implemented several days earlier than the start of red alerts. The results proved that the implementation of emission reduction measures 1–2 days before red alerts could lower the peak of PM_2.5_ concentrations significantly. Given the difficulty of precisely predicting the duration of heavy pollution episodes and the fact that successive heavy pollution episodes may return after red alerts, emergency measures should also be implemented one or two days after the red alerts.

## Introduction

Beijing, located in the Northern China, is one of the most populous cities in the world. Beijing is the political, cultural, and educational center of China. In the past decade, air quality in Beijing has been improved notably through effective air pollution prevention and control measures^1–3^. However, the total discharge of air pollutants remains much larger than the environmental capacity of Beijing, which easily leads to heavy pollution episodes under unfavorable weather conditions^4^.

To effectively implement emergency measures, mitigate serious air pollution episodes, and protect public health, Beijing Municipal Government launched “Heavy Air Pollution Contingency Plan” in 2013. According to the predicted seriousness and duration of air pollution episodes, air pollution alerts in Beijing are categorized into four levels, which are, blue, yellow, orange, and red alerts (with a corresponding alert level of four, three, two, and one). Each type of alerts has its unique emergency measures. In 2015, Beijing revised the contingency plan to better deal with the air pollution episodes^5^. Air pollution levels are determined based on the duration of air pollution episodes at the hour, rather than the day level, which brings extra difficulties in properly setting the starting point and lowers the thresholds of air pollution alerts (Supplementary Table 1). For each alert level, specific instructions for health protection and mandatory emergency measures are given accordingly. The red alert is the most stringent level of air pollution alerts and predicts air pollution episodes that will last for more than 72 hours. Its mandatory emergency measures mainly include suspending courses of primary and middle schools, implementing the odd–even license plate policy, stopping outdoor construction work, and banning fireworks and outdoor barbecues. According to the revised contingency plan, Beijing released two red air pollution alerts in December 2015, which aroused wide attentions and was selected as one of the top ten major episodes in the environmental protection field in China^6^.

Due to its significant influences in China, many international events are hosted here every year. Therefore, air pollution in Beijing, especially frequent air pollution episodes, not only affects local residents’ life, but also attracts international attentions. As one major city in China that suffers from serious air pollution, the air quality in Beijing has naturally received massive emphasis.

From 2013 to 2015, Beijing, as well as other heavily polluted cities^7, 8^, experienced several severe air pollution episodes during winter, raising considerable public attention. Most studies of air pollution have focused on the variations and evolutions of chemical composition and correlations between meteorological factors and air pollution^9–11^. Several studies revealed that formation mechanisms and causes of severe air pollution episodes were mainly attributed to three aspects: (1) stable synoptic meteorological conditions; (2)secondary chemical reactions; (3) regional transport of airborne pollutants^12, 13^. However, these studies mainly focused on the evolutions of air pollution episodes without the implementation of emission reduction measures whilst few studies have evaluated the effects of air pollution alerts and corresponding emission reduction measures. Several studies^14, 15^ have examined the characteristics and effects of emergency measures on mitigating heavy air pollution during mega events. Some studies have been examined to understand variations of airborne pollutants and effects of emergency measures on air quality during major international activities, such as the Beijing Olympic Game^3, 16, 17^, the 21th Asia–Pacific Economic Cooperation(APEC) conference^18^, 2010 Shanghai Expo and the 2011 Guangzhou Asian Gamesand the 2011 Universiade^19–21^. Most of these studies explained the influence of atmospheric compositions or meteorological conditions on local air quality under much better weather conditions. Nevertheless, few studies quantitatively examined the relationships between the emissions and concentrations of ambient air pollutants during heavy air pollution episodes.

By analyzing possible origins^22^, chemical components^23^ and seasonal variations^24–27^ of airborne pollutants, characteristics of the air quality in Beijing has been examined from different perspectives. Furthermore, temporary measures for emission reduction implemented during the two red alerts provide us with rare and valuable data to understand the effects of emission reduction on the variations of local air quality. By combining the monitored air quality data with air quality prediction models, we attempted to address the following questions for the air pollution episodes (1) spatial and temporal characteristics of PM_2.5_ during two typical air pollution episodes that experienced sharp increase and nonunified distribution of PM_2.5_ concentrations; (2) quantitative effect evaluation of emission reduction measures on local PM_2.5_ concentrations during two red alerts; (3) Suggestions for air quality prediction and management. This research provides important reference for properly evaluating air pollution alerts. Furthermore, useful suggestions are given for better implementing future air pollution alerts and corresponding emission reduction measures in China.

## Results

### Temporal Variations of PM_2.5_ concentrations

In 2015, annually averaged PM_2.5_ concentrations at 35 sites in Beijing was 80.6 ± 71.8 μg · m^−3^, about 1.5 times higher than the threshold (35 μg · m^−3^) required by the new air quality standard^28^. There were 42 heavily polluted days with an average concentration of 238.6 ± 70.6 μg · m^−3^, accounting for about 11.5% of the total days in 2015. Air pollution episodes in Beijing mainly occurred in the cold months with provided central-heating (November, December, January, February, and March), accounting for 83% of the total heavily polluted days. However, severe air pollution may also be witnessed in June and July when the emission intensity decreased and meteorological dispersion conditions improved significantly^22^.

**Figure 1:**
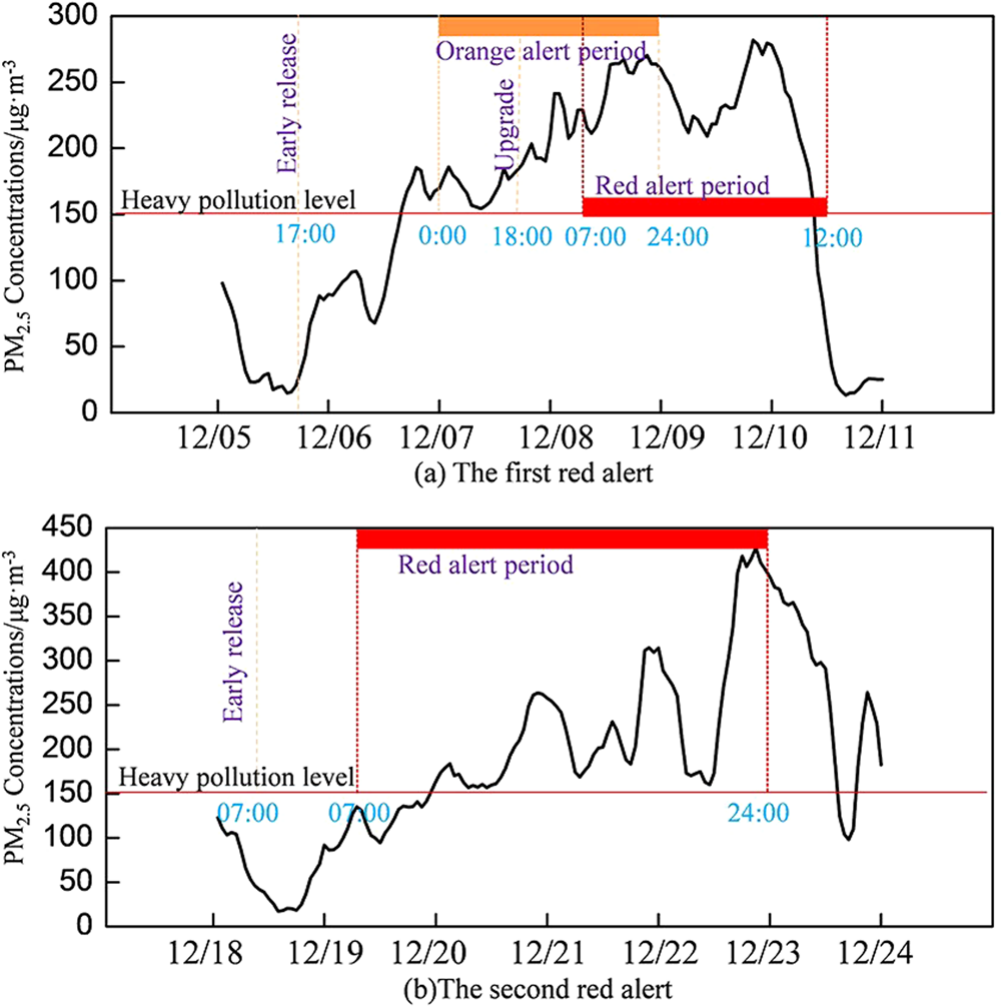
Temporal variations of hourly averaged PM_2.5_ concentrations at 35 stations in Beijing during two red alerts.

In 2015, Beijing released eight blue alerts (9 days in total), seven yellow alerts (20 days in total), two orange alerts (5 days in total). In December 2015, Beijing released two red air pollution alerts. The first red alert started from at 07:00, December 8^th^ and ended at 12:00, December 10^th^; the second one started at 07:00, December 19^th^ and ended at 24:00, December 22^th^ (Beijing local time). Before the launch of the first red alert (Fig. 1), the emergency office released an orange alert 31 hours in advance at 17:00, December 5^th^, when the general air quality in Beijing remained good. Although the office set the orange alert from 09:00 on December 6^th^ to 24:00, December 9^th^, the present alert was upgraded to the red level at 18:00, December 7^th^ based on the latest forecast. During the first red alert, hourly averaged PM_2.5_ concentrations slowly increased to the peak of 290 μg · m^−3^ at 21:00, December 9^th^. Influenced by the cold air front and northerly wind, PM_2.5_ concentrations decreased significantly after 12:00 December 10^th^, indicating the end of this air pollution event. The total alert period (orange and red) lasted for 84 hours.

The second red air pollution alert was released 24 hours in advance, and lasted from 07:00, December 18th to 24:00 on December 22 (approximately 90 hours in total). The hourly averaged PM_2.5_ concentration kept higher than 150 μg · m^−3^ from December 19^th^ to December 22^th^. The highest hourly PM_2.5_ concentration was 421 μg · m^−3^ which occurred at 20:00, December 22^nd^; then it declined slowly on the morning, December 23^rd^. However, with the end of the red alert, the variation of air quality in the northern and southern part of Beijing, demonstrated notable differences. The air quality in the northern areas had been improved to moderately polluted, whereas the air quality in the city center and southern areas remained heavily polluted. This phenomenon may be attributed to the weak high-pressure system affecting the northern parts of Beijing. Since 12:00, December 23^rd^, high PM_2.5_ concentrations returned and again aggravated the air quality in the northern areas, resulting an rapidly increased hourly averaged PM_2.5_ concentration from 90 μg · m^−3^ to 260 μg · m^−3^. However, considering the complexity of meteorological conditions, social endurance, and work arrangement, it is presumed that the general air quality should be improved notably since the morning, December 23^rd^ and thus the previously set duration for this red alert was not extended. As a result, severe air pollution after this red alert caused negative influences on people’s daily life and health.

### Spatial Variations of PM_2.5_ concentrations

**Figure 2:**
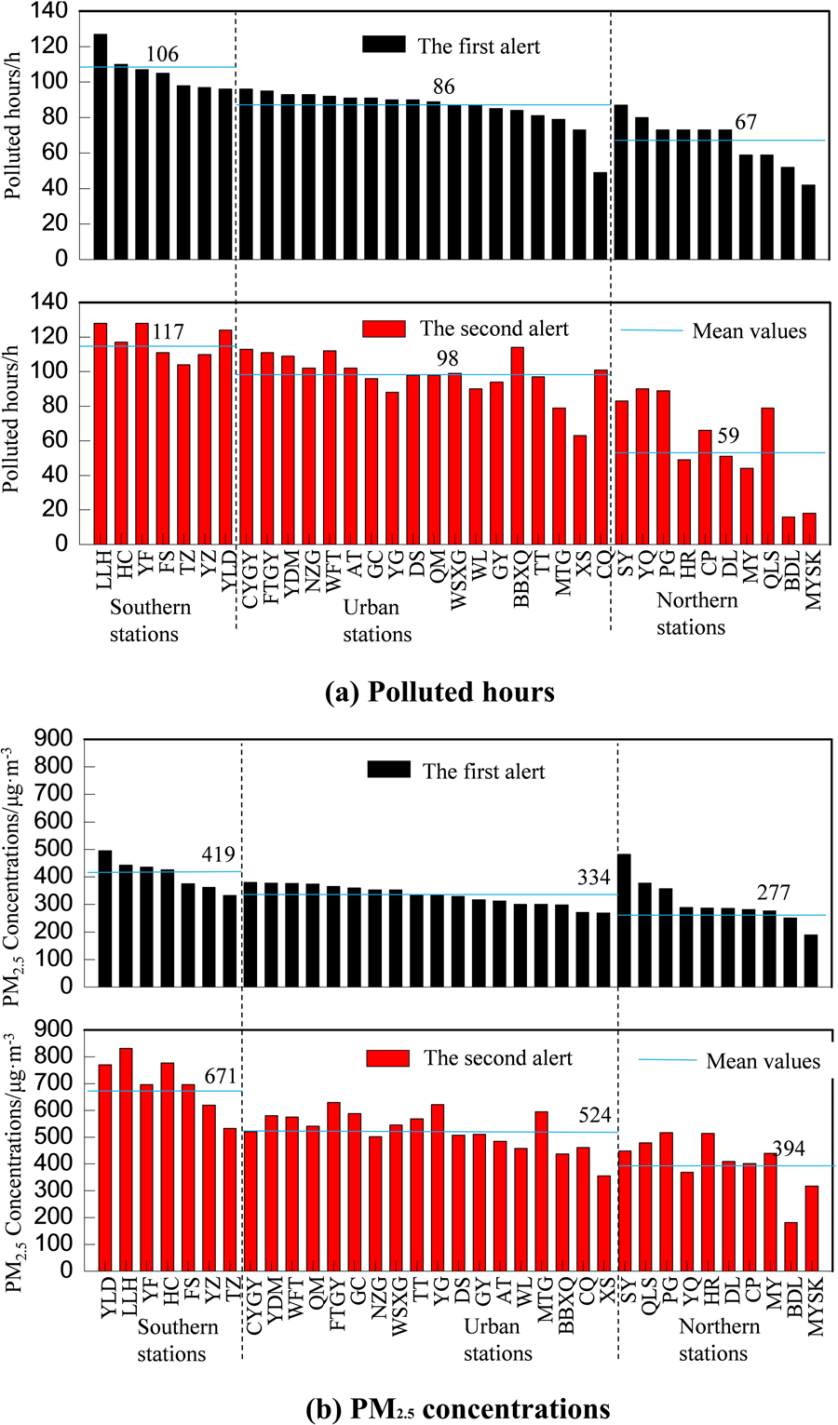
(**a**) The number of heavily polluted hours and (**b**) extreme values of hourly averaged PM_2.5_ concentrations at different stations in Beijing during two red alerts.

During the first red alert, the highest hourly averaged PM_2.5_ concentration 496 μg · m^−3^ was witnessed at the Yong Ledian (YLD) station, southeast of Beijing while the highest hourly averaged PM_2.5_ concentration 831 μg · m^−3^ during the second red alert occurred at the Liu Lihe (LLH) station, southwest of Beijing (Fig. 2). Notably, PM_2.5_ concentrations at each site during the second red alert were much higher than that during the first red alert. Meanwhile, PM_2.5_ concentrations within different parts of Beijing, especially the southern and northern parts, varied significantly. For each station, the duration of heavy air pollution ranged from the shortest 10 hours to longest 130 hours in both two red alerts. According to accumulative hours of heavy air pollution, notable spatial patterns of PM_2.5_ concentrations were found: PM_2.5_ concentrations in southern stations >PM_2.5_ concentrations in urban stations >PM_2.5_ concentrations in northern stations.

To better understand the spatial variations of PM_2.5_ concentrations across Beijing during two red alerts, detailed analysis was conducted for three representative stations, the YF, JCZX and DL station.

**Figure 3:**
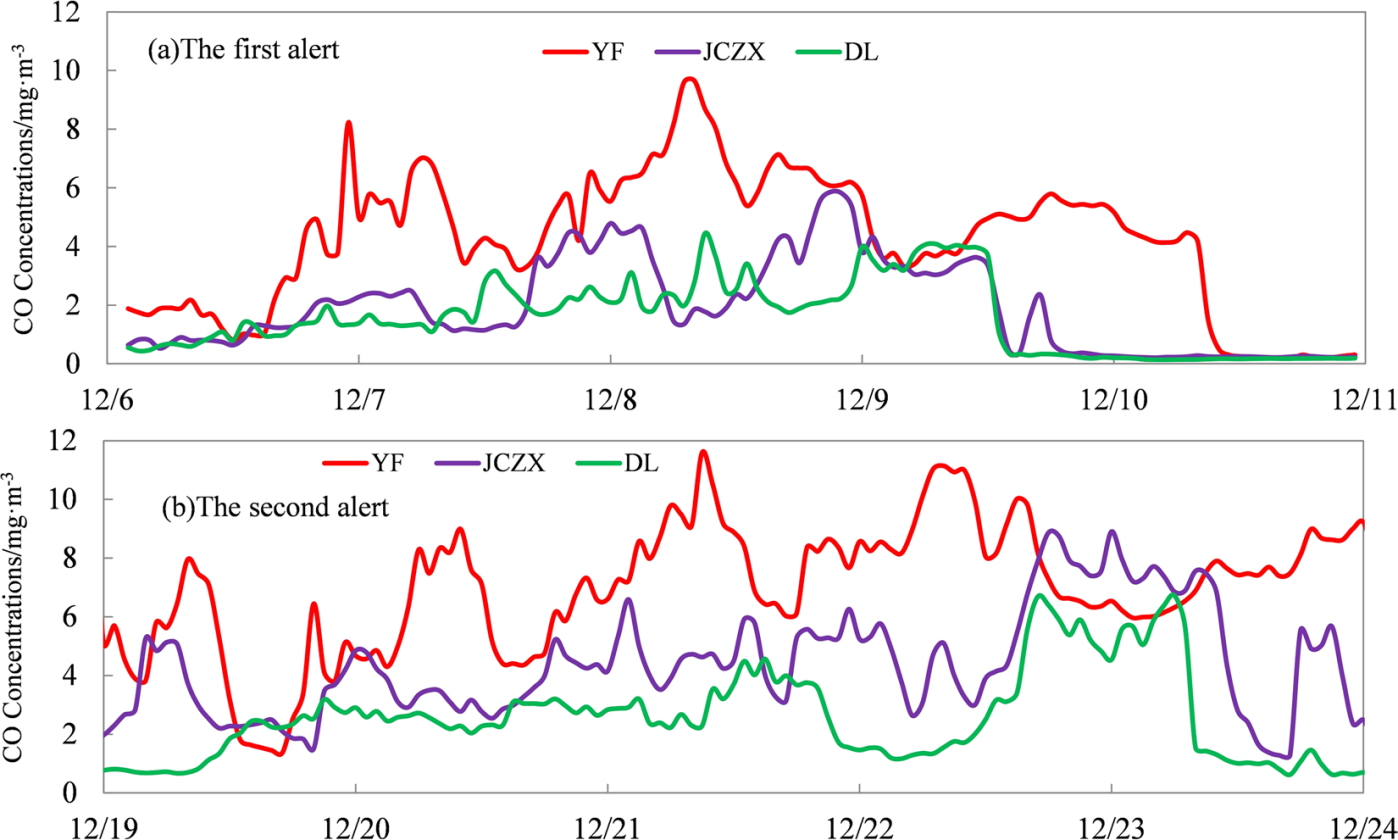
Variations of hourly averaged CO concentrations at 3 representative stations in Beijing during two red alerts.

The life time of CO in the atmosphere is between 1 and 2 months. In case of serious pollution episodes, CO is usually in a stable state and can be used as a tracer to analyze the transportation of pollutants in the atmospheric environment. According to Fig. 3, it was noted that the peak concentration of CO occurred in turn in the YF, JCZX and DL station. Similar to the spatial variations of CO concentration, at the initial stage of red alerts, high PM_2.5_ concentrations (PM_2.5_ > 150 μgm^−3^) mainly occurred in the southern stations; as the air pollution process continued, PM_2.5_ concentrations in central and the northern parts of Beijing increased rapidly, indicating a clear south-to-north transportations trend of airborne pollutants. At the end stage of the air pollution episodes, PM_2.5_ concentrations in the northern parts of Beijing started to decrease rapidly and PM_2.5_ concentrations in the southern parts dropped successively, demonstrating an north-to-south pattern for the air quality improvement process.

During the two red alerts, the northern and western parts of Beijing are of higher elevation with more strong winds, which are favorable conditions for the dispersion of airborne pollutants. Meanwhile, the southern parts of Beijing are surrounded by hills and have poor winds, which are favorable for the accumulation, and unfavorable for the dispersion of airborne pollutants. The main reason for the notable variations of PM_2.5_ concentrations across Beijing is the geographical and meteorological conditions^24, 25^. Additionally, the air quality in the southern parts of Beijing is more likely to be influenced by regional transportation of airborne pollutants from neighboring cities.

In addition to the geographical and meteorological conditions, and regional transportation of airborne pollutants, coal consumption, especially the residential emission in the southern areas, also contributed to the non-unified PM_2.5_ distributions during the two red alerts. In Beijing, factories, development zones and rural areas, where direct combustion of solid fuel in low-efficiency stoves, are mainly concentrated in the southern parts of Beijing. Meanwhile, the use of coals in the northern parts of Beijing has been reduced significantly by such fuels as natural gas, which produced less airborne pollutants.

Hopane can be used to distinguish the maturity and type of fossil fuels due to its stable nature and indicative content. Meanwhile, the ratio of C30 to C31R in hopane can be used to distinguish organic compounds from coal combustion or vehicle emissions^29^. Previous studies^30, 31^ suggested that the ratio of C30 to C31R from gasoline and diesel combustion was generally larger than 2.5, whilst the ratio of C30 to C31R from coal combustion ranged between 0.1 and 2.5. E.g., the ratio of C30 to C31R for Honeycomb, Briquettes, bituminous coal was 0.88, 0.99 and 0.72 respectively. In this study, we analyzed the ratio of C30 to C31R based on samples of airborne pollutants collected at the three representative stations and the results are demonstrated as Table 1. The ratio of C29 to C31R for three stations ranged between 0.70~0.90 during two red alerts, and the C29/C31R value for the YF station was the largest. The variations of C29/C31R values across Beijing indicated that the contribution of coal combustion to high PM_2.5_ concentrations in southern areas was much higher than that in the central and northern areas. In addition to C29/C31, CO is a indicator for the incomplete combustion of fuels. According to the variations of C29/C31 and CO, the incomplete combustions of coals contributed substantially to the deterioration of air quality during the red alerts.

**Table 1:** Statistics of C29/C31R, CO concentrations and PM_2.5_ concentrations at three stations in Beijing during two red alerts.

	C29/C31R			CO/mg · m^−3^			PM_2.5_/ug · m^−3^		
	YF	JCZX	DL	YF	JCZX	DL	YF	JCZX	DL
The first alert	0.84	0.75	0.70	5.51	2.67	2.14	273.0	222.6	199.0
The second alert	0.90	0.81	0.76	7.60	4.80	2.80	415.0	262.0	182.0
Annual average	0.61	0.55	0.45	1.60	1.30	0.90	100.1	82.2	64.6

In the future, as Liu *et al*.^32^ pointed out, the reduction of residential emissions in southern areas, could be implemented through such means as replacing coal fuels with electricity or natural gas sources. The change of fuel materials can lead to notable improvement of air quality in Beijing. Meanwhile, notable spatial variations of PM_2.5_ concentrations across Beijing during the heating season may be reduced as well.

### Variations of PM_2.5_ components

**Figure 4:**
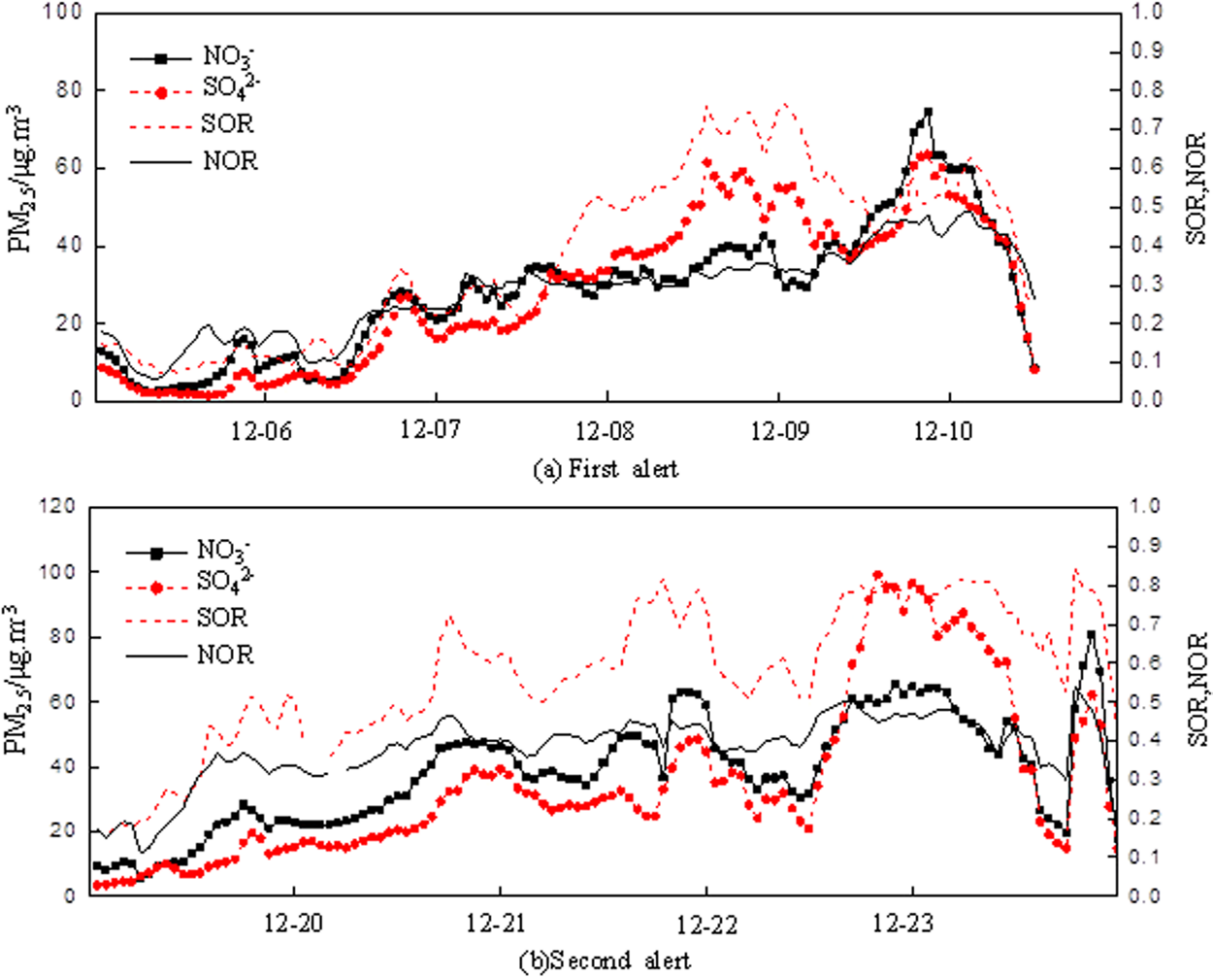
Variations of SO_4_^2−^, NO_3_^−^, SOR, NOR during two red alerts periods at JCZX site in Beijing in 2015.

We further caculated the variations of major chemical components in PM_2.5_ at the JCZX site in Beijing during both red alert periods. Average mass concentrations of NO_3_^−^, SO_4_^2−^, NH_4_^+^ were (37.76 ± 11.74), (40.42 ± 13.36), and (36.45 ± 7.65) μgm^−3^ respectively during the first red air alert, whilst average mass concentrations of these components changed to (43.90 ± 13.91), (41.64 ± 24.34), and (34.03 ± 14.03) μgm^−3^ during the second case. NO_3_^−^, SO_4_^2−^ and OC were the three species with the highest concentrations, whilst Cl^−^, K^+^, Ca^2+^ were the three species with lowest concentrations. Concentration of NO_3_^−^ accounted for 23% of average PM_2.5_ concentrations during the first red alert, and accounted 25% during the second one. Different from other air pollution episodes, the concentration of NO_3_^−^ was much higer than SO_4_^2−^. The ratio of NO_3_^−^/SO_4_^2−^ was 0.93 and 1.05, respectively during two red alerts, which were 1.96 and 2.22 times higher than the annual averaged value of 0.48. The higher NO_3_^−^/SO_4_^2−^ ratio indicated that the motor vehicles played a more important part during the second alert^10, 11^. In addittion, the SOR (sulfur oxidation ratios) and NOR (nitrogen oxidation ratios) were introduced into this research (Fig. 4). The SOR was much higher during the second alert. Therefore, although the concentration of SO_4_^2−^ was lower than that of NO_3_^−^, the oxidation rate from SO_2_ to SO_4_^2−^ was faster. This phenomenon may be attributed to the existence of high level of Nox, which accelerated the reaction from SO_2_ to SO_4_^2−^. Therefore, nitrogen oxides emitted by motor vehicles can accelerate the generation of secondary ions, and further deteriorate air quality^12, 13^.

### Effects of emission reduction measures on PM_2.5_ concentrations

Compared with other heavily polluted days (excluding those polluted days during two red alerts) in 2015, the ratio of PM_2.5_ to SO_2_, CO and NO_2_ (Table 2) all decreased during two red air alerts, indicating that concentrations of secondary air pollutants (e.g. PM_2.5_), dropped much faster than that of primary pollutants such as SO_2_ and CO. The ratio of NO_2_ to CO also experienced significant decrease, which was mainly attributed to the limitation of vehicle emissions through the odd-even license plate policy.

**Table 2:** Ratios between different air pollutants during different heavily polluted episodes in Beijing.

Airborne pollutants	Stations	During other heavily polluted days	During the first alert	During the Second alert
PM_2.5_/SO_2_	JCZX	16.705	8.269	8.697
	DL	15.043	11.314	11.385
	YF	8.033	15.243	27.141
PM_2.5_/CO	JCZX	0.080	0.056	0.053
	DL	0.082	0.063	0.070
	YF	0.066	0.054	0.052
PM_2.5_/NO_2_	JCZX	2.593	2.233	2.097
	DL	3.565	2.790	3.035
	YF	3.622	3.662	3.708
SO_2_/CO	JCZX	0.011	0.008	0.008
	DL	0.011	0.008	0.012
	YF	0.008	0.004	0.002
NO_2_/CO	JCZX	0.032	0.028	0.027
	DL	0.026	0.023	0.025
	YF	0.018	0.015	0.014
PM_2.5_ accumulation rate/μg · m^−3^ · h^−1^	JCZX	5.6	1.8	3.9
	DL	5.1	1.6	3.5
	YF	5.8	5.7	5.8

**PM**_**2.5**_ **accumulation rate** = (Max PM_2.5_ concentration- 150 μg · m^−3^)/polluted hours.

Compared with other heavily polluted days, the accumulation rate of PM_2.5_ concentrations was much smaller during the two red alerts. Despite unfavorable weather conditions for the diffusion of air pollutants, concentrations of main air pollutants remained declined, indicating that emergency measures for regional emission reduction worked effectively. Although it is unlikely for these measures to improve local air quality significantly (e.g. from heavy pollution to moderate or slight pollution instantly), they performed effectively in slowing the accumulation rate of PM_2.5_ concentrations and lowering the peaks of PM_2.5_ concentrations during severe air pollution episodes^33, 34^.

**Figure 5:**
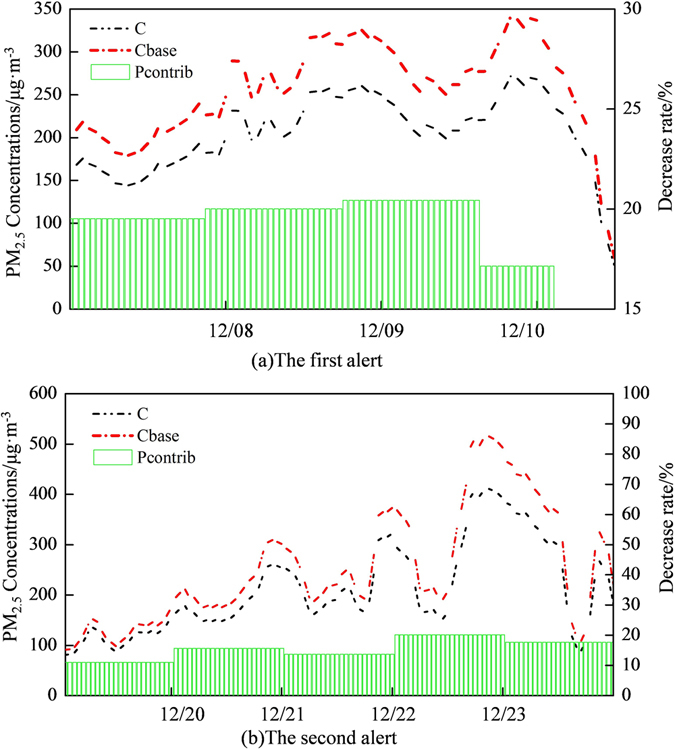
Effects of emission reduction measures on PM_2.5_ concentrations during two red alerts (*P*_*contrib*_, *C* and *C*_*base*_ are the contribution rate of emission reduction to PM_2.5_ concentrations, the simulated PM_2.5_ concentration under the emission reduction scenario and simulated PM_2.5_ concentration in baseline scenario respectively).

According to the simulation results of CAM*x* (Fig. 5), it is note that emergency measures implemented during the first red alert lowered daily PM_2.5_ concentrations by 19.53%, 20.01%, 20.44% and 17.16% respectively (Dec 7~Dec 10, 2015) whilst the lowered daily PM_2.5_ concentrations during the second red alert was 11.08%, 15.62%, 13.72%, 20.20% and 17.71% respectively (Dec 19~Dec 23, 2015). The first red alert was released based on an enhanced orange alert. Affected by earlier implementation of emission reduction measures, the decreasing rate of PM_2.5_ concentrations was larger than that during the second red alert. Moreover, the emergency measures decreased the peak of PM_2.5_ concentrations by about 10% to 20%, which significantly reduced the negative influences of PM_2.5_ on locals’ health and daily life. Though the implementation of regional emission reduction measures during red alerts, even if we could not change the evolution process of heavy pollution episodes, these measures indeed mitigated extremely high PM_2.5_ concentrations.

In the current scenario, emergency measures were implemented from the starting point of two red alerts. In this research, we further examined the potential effects of emission reduction measures on PM_2.5_ concentrations if these measures were implemented days before the red alerts. Potential effects of emergency measures implemented in advance are presented as Table 3. According to Table 3, the implementation of emission reduction measures 2–3 days before red alerts could lower the peak of PM_2.5_ concentrations (31~36%) and lower the daily averaged PM_2.5_ concentrations (19~22%) significantly. Compared with the scenario with 3 days’ in advance, emission reduction measures conducted four days before red alerts made limited extra contribution to the reduction of PM_2.5_ concentrations. Furthermore, the reduction effects of PM_2.5_ concentrations conducted two or three days before red alerts were of slight differences. Given the high cost and difficulties of implementing emission reduction measures, we suggest that the most appropriate time for executing emergency measures should be 24–48 hours before the period with a rapid rise of PM_2.5_ concentrations^35^. In this case, substantial decrease of PM_2.5_ concentration peaks can be realized with affordable social and economical costs.

**Table 3:** Effects of implementing emission reduction measures of four, three, two, one and zero days before the start of red alerts on the reduction of PM_2.5_ concentrations.

Emission scenarios (Reduction measures implemented)	PM_2.5_ peaks (%)				Daily averaged PM_2.5_ concentration (%)			
	YF	JCZX	DL	Average	YF	JCZX	DL	Average
4 days	37	40	38	38	25	22	23	23
3 days	36	38	34	36	23	20	22	22
2 days	33	32	29	31	20	18	20	19
1 day	27	26	22	25	19	16	19	18
0 day	16	15	12	14	7	6	7	7

Naturally, the more emission reduction measures taken, the more cost will be incurred. Cost–benefit analysis (CBA) is a widespread tool to support decision-making. However, pollutant concentration alleviation cost curves were rarely employed in previous studies^36^. Furthermore, in practice, limited regional environmental management cooperation has been implemented in China, due to the lack of institutional arrangements during the major national and international events (i.e., Beijing during the 29th Olympic Games in 2008, Shanghai during the 2010 World Expo, Guangzhou during the 16th Asian Games in 2010, and Shenzhen during the 26th Universidade in 2011)^37^. Hence, for the next air quality assurance work, regional environmental management departments should further strengthen the cost-effectiveness analysis of emission reduction measures, especially during major events and air pollution alerts.

## Discussions

Based on observed data, we examined the temporal and spatial variations of PM_2.5_ concentrations in Beijing during two red air pollution alerts in 2015. The results demonstrated that PM_2.5_ concentrations varied significantly across Beijing during heavy pollution episodes. Generally, PM_2.5_ concentrations in the southern parts of Beijing were much higher than those in the northern parts during both red air alerts. Additionally, air pollution episodes occurred earlier in the southern parts and the dispersion of airborne pollutants started in the northern parts. The first red alert was set based on the upgrade of an orange alert. Although the second red air pollution alert was released 24 hours in advance, it was not further extended to the afternoon, December 23^rd^, when high PM_2.5_ concentrations returned and deteriorated air quality in Beijing again.

Non-unified PM_2.5_ distributions across Beijing were a major reason for prediction errors. A diversity of factors, including the inaccurate or incomplete emission inventories, rapidly changing meteorological conditions, complicated influences of meteorological influences on airborne pollutants, and regional transports of airborne pollutants, may as well lead to biased prediction results. In terms of potential uncertainties in emissions, the residential emissions have been overlooked in air pollution control strategies whilst major emphasis has been put on the industrial and vehicle emissions^34^. In terms of changing meteorological conditions, the complex small-scale flow field, the abrupt wind direction and specific topographical conditions all resulted in non-unified PM_2.5_ distributions, which may not be precisely simulated. Furthermore, a comprehensive understanding of complicated interactions between meteorological factors and PM_2.5_ concentrations remains challenging^26^. Although our previous research^25, 26^ examined the influence of individual meteorological factors on PM_2.5_ concentrations, comprehensive influences of all meteorological factors were not quantified, causing extra difficulties in predicting the trend of PM_2.5_ concentrations. Additionally, regional transport constantly plays a key role in affecting local PM_2.5_ concentrations. According to a comprehensive source apportionment method (http://www.bjepb.gov.cn/bjepb/323265/340674/396253/index.html), regional transport of PM_2.5_ accounts for nearly a third of the annually averaged PM_2.5_ concentration in Beijing whilst this rate further increased to approximately 47%–78% during heavily polluted days^38^.

Emergency measures implemented during two alerts varied in specific terms and there were notable differences between durations for Beijing and its surrounding areas. Through the CAM*x* model, we calculated that the emission reduction measures cut down 10–30% of the total emissions of air pollutants and reduced the peaks of PM_2.5_ concentrations by 10–20%. Furthermore, we simulated the scenarios of implementing emergency measures days before the period of red alerts. Considering the effects of emergency measures on the reduction of PM_2.5_ concentrations and corresponding costs, we suggest that emergency measures should be implemented 24–48 hours before red alert periods.

The severe air pollution, without the mitigation of any further alert and emergency measure, caused extra threats to people’ health. For better predicting the variation trend of air pollutants and set proper air pollution alert levels and durations, as well as corresponding emergency measures, more information should be comprehensively considered. However, many challenges remain. Given the difficulty of precisely predicting the duration of heavy pollution episodes and the fact that severe heavy air pollution episodes may return after red alerts, the emergency measures should be implemented not only several days before, but also one or two days after the red alerts.

During the Asia-Pacific Economic Cooperation (APEC) summit (1–12 November, 2014) and the Parade on the 70th Victory Memorial Day for the Chinese People’s War of Resistance against Japanese Aggression (PARADE) (20 August-3 September, 2015), Beijing and its surrounding cities jointly implemented emergency measures with increased intensity to reduce emissions in advance and the monitoring data proved the improvement of air quality^39^. The regional air quality protection measures were taken several days before the opening ceremony. Based on the prediction results, cities to the southwest and southeast of Beijing were informed to take measures accordingly, which not only led to a good reduction effect and reduced social costs. As a result, the experiences of APEC and PARADE can further guide the release and implementation of red alerts. Due to notable differences in geographical and meteorological conditions and PM_2.5_ concentrations, we suggest that emergency emission reduction measures for different parts within Beijing should be proposed and implemented accordingly. Meanwhile, it is noted that early warning standard and emergency measures were of large differences across cities. Hence, for the next-round revision of the regional heavy air pollution contingency plan, advanced approaches for setting unified criteria of alert levels and corresponding emergency measures should be employed comprehensively in the Beijing-Tianjin-Hebei region.

To fully consider the regional transport of airborne pollutants and better predict local and regional air quality, a regional, instead of local, air quality forecast system for the entire Beijing-Tianjin-Hebei region should be established. In this case, local governments within this region may work together to decide when and how emergency measures should be implemented simultaneously during regional air pollution alerts. The release of regional joint air pollution alerts and their corresponding contingency plans is key to the improvement of local and regional air quality in the Beijing-Tianjin-Hebei region. Since the interactions between meteorological factors and PM_2.5_ concentrations are highly complicated and severe air pollution episodes can occur after red alerts, the duration of emergency measures may be extended accordingly.

Currently, the evolution, genetic analysis, prediction and evaluation of air pollution, are generally investigated separately, yet these factors are closely linked. For future research, scholars from different background should work together for better understanding air pollution episodes and managing local and regional air quality. Meanwhile, in addition to red air pollution alerts, we should place more emphasis on the evaluation of emergency measures implemented during blue, yellow and orange alerts. This research not only proposes an effective approach for evaluating emergency measures implemented during previous air pollution alerts, but also provides useful suggestions for better designing and implementing future contingency plans.

## Materials

### Measurement data of airborne pollutants

**Figure 6:**
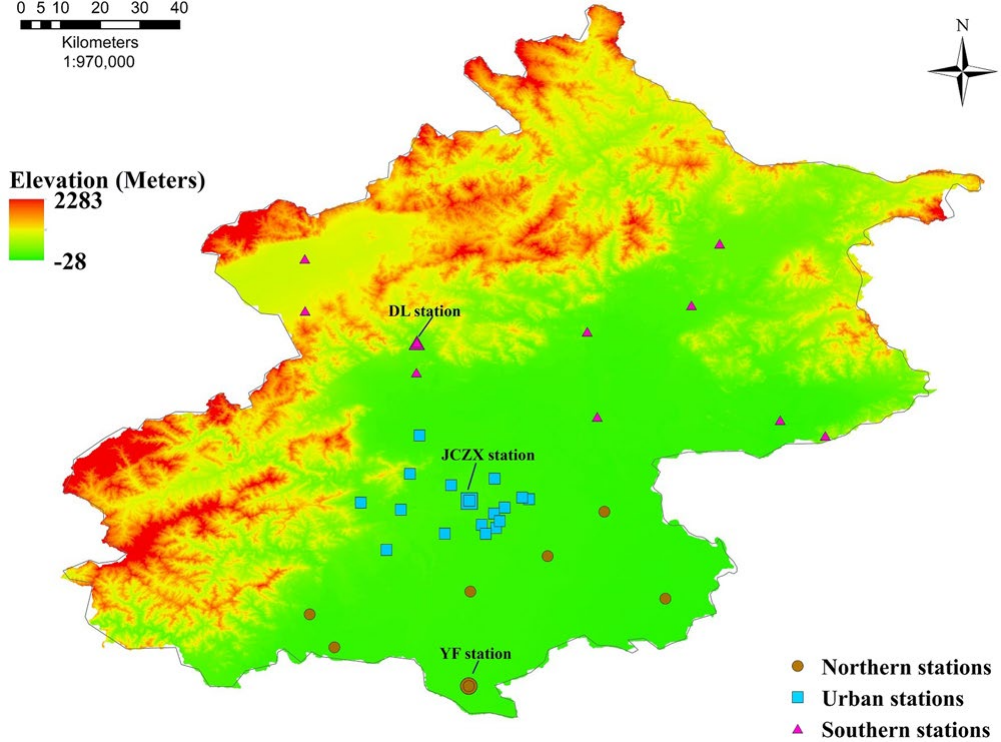
Categories and distributions of air quality observation stations in Beijing. This Map was generated using ArcGIS, Version 10.3 (www.esri.com/software/arcgis).

In addition to PM_2.5_, we monitored other airborne pollutants for a better understanding of the characteristics of air pollution evolutions during the two red alerts. Airborne pollutants in Beijing are monitored at 35 stations (Fig. 6 **)**. PM_2.5_ is recorded using the thermo 1405F instrument and SO_2_ is monitored using a Thermo Fisher 43I pulsed UV fluorescence analyzer. Thermo Fisher 42C and Thermo Fisher 48C (Thermo Fisher Corporation, USA) are used to measure NO/NO_2_/NOx and CO respectively. Among these 35 sites, the DL station is located in the northern part of suburban areas of Beijing; the JCZX station is located in urban areas between the 2^nd^ ring and 3^rd^ ring road within Beijing; The YF station is close to the southern boundary of Beijing. Since there are more monitoring instruments with better operation and maintenance in the three stations, the DL, JCZX and YF station are selected as the representative station for monitoring airborne pollutants in the northern, urban and southern of Beijing. We employed dichloromethane and methanol (3:1) as solvent ultrasonic to remove the insoluble particles and extract PM_2.5_ samples collected using quartz films at three sites (the DL, JCZX and YF stations). The extract was concentrated to 1 ml through rotary evaporation with the blowoffing of high-purity nitrogen. Organic compounds were determinated using the Agilent GC-MS (6890Plus/5953N). The PM_2.5_ water-soluble ionic content was monitored at the JCZX station using the 9000B Ambient Ion Monitor (URG Corp., Chapel Hill, NC, USA), which has two chemical analysis systems, including a ICS-90 (Dionex Corp., Sunnyvale, CA, USA). The real-time weather charts were downloaded from Korea Meteorological Administration (http://web.kma.go.kr/chn/weather/images/analysischart.jsp) to analyze the weather conditions.

### Models simulation and scenarios

We employed the WRF-CAM*x* model for simulating the effects of emission reduction and detecting the optimal time to start air pollution alerts. The mesoscale meteorology model (i.e., WRF) was used to provide the meteorological field, and the CAM*x* model was used to simulate the variation of airborne pollutants^40^. CAM*x* has been widely used as an effective tool for simulating processes of air pollution^41, 42^. In this research, CAM*x* was set with a 12 km grid resolution that covered most areas in the East Asia (including Japan, South Korea, China, North Korea, and other countries). The vertical layer was divided into 20 unequal layers, 8 of which had a distance of less than 1 km to better describe the layer structure of atmospheric boundary. PM_2.5_ in CAM*x* was simulated based on several physical and chemical mechanisms, including (1) horizontal advection scheme (PPM), (2) implicit Euler vertical convection scheme, (3) horizontal diffusion of K theory, (4) Saprc99 gas-phase chemical mechanism, and (5) EBI calculation method. Initial and boundary conditions for air quality simulations were generated using the default CAM*x* profiles. The simulation period was set during December 1^st^, 2015 to December 31^st^, 2015, and a spin-up period of 5 days was set to eliminate the influence of uncertain initial conditions.

Two simulation scenarios, including a baseline emission scenario and an emission reduction scenario, were set up to evaluate the effects of emission reduction measures on PM_2.5_ concentrations under the same meteorological field. The calculation formula was as follows:

1 $${P}_{contrib}=\frac{C-{C}_{base}}{C}\times 100 \% $$

where *P*_*contrib*_, *C* and *C*_*base*_ are the contribution rate of emission reduction to PM_2.5_ concentrations, the simulated PM_2.5_ concentration under the emission reduction scenario and simulated PM_2.5_ concentration in baseline scenario respectively. To better understand the influence of emission reduction measures on PM_2.5_ concentrations and find the optimal starting point for the air pollution alerts, we further defined five emission scenarios, which indicate emission reduction measures started at 5 different time points (Zero, One, Two, Three and Four days in advance of the alerts). The intensity of emission reduction in these scenarios were set consistent with red alerts (Table 4, and more details in Supplementary Table 2).

**Table 4:** Daily averaged emission reductions of SO_2_, NO*x*, PM_2.5_, PM_10_, and VOCs in the Beijing-Tianjin-Hebei region.

Airborne pollutants			SO_2_	NO*x*	PM_10_	PM_2.5_	VOCs
Beijing	Red alert	reduction emission/t	15	182	454	45	200
		reduction rate/%	14	32	67	25	29
	Orange alert	reduction emission/t	14	118	381	32	142
		reduction rate/%	13	21	57	18	21
Tianjin	Red alert	reduction emission/t	80	220	180	79	337
		reduction rate/%	12	21	28	25	26
	Orange alert	reduction emission/t	44	37	139	59	210
		reduction rate/%	7	4	21	18	16
Hebei	Red alert	reduction emission/t	665	1831	1378	735	1767
		reduction rate/%	22	37	28	28	28
	Orange alert	reduction emission/t	442	1300	1026	534	1310
		reduction rate/%	14	27	21	20	21

During the periods of air pollution alerts in Beijing, some cities surrounding Beijing started up different levels of air pollution alerts as well according to their specific air pollution contingency plans. These emergency measures included the odd-even license plate policy, 30% emission reduction from several heavy-polluting factories, cleaning the roads, and shutting down most construction manufacturers. The reduction of anthropogenic emissions was calculated based on the concentrations of SO_2_, NO*x*, PM_2.5_, PM_10_, and VOCs in the Beijing-Tianjin-Hebei region. According to a bottom-up investigation of thousands of individual pollution sources including power plants, industrial enterprises, and heating boilers^43^, the total emission for each city was calculated respectively. Grid-based emissions from vehicles were calculated based on micro-scale vehicle activities and speed-dependent emission factors^44^. Results showed that during red air pollution alerts, the daily emission reduction of SO_2_, NO*x*, PM_10_, PM_2.5_, and VOCs in Beijing was 15 t, 182 t, 454t, 45t and 200t, accounting for 14%, 32%, 67%, 25%, and 29% of the total emissions as usual in Beijing. Emergency measures led to 25%~30% emission reduction of SO_2_, NO*x*, PM_2.5_, PM_10_, and VOCs in the Beijing-Tianjin-Hebei region during the two red alerts. In this case, remained air pollutant emissions could be imported to the model for simulating the variations of PM_2.5_ concentrations and examining the relationship between air pollutant emissions and ambient concentrations.

Emissions of SO_2_ and PM were estimated using a mass-balance approach expressed by Eqs 2 and 3, respectively. NOx and NMVOC emissions were calculated using an emission factor methodology expressed by Eq. 4.

2 $${{\rm{E}}}_{{{\rm{SO}}}_{2}}=\sum _{{\rm{i}},{\rm{j}},{\rm{k}},{\rm{m}}}{{\rm{A}}}_{{\rm{i}},{\rm{j}},{\rm{k}},{\rm{m}}}\times {{\rm{Scont}}}_{{\rm{i}},{\rm{m}}}\times (1-{{\rm{Sr}}}_{{\rm{i}},{\rm{j}},{\rm{k}},{\rm{m}}})\times (1-{{\rm{\eta }}}_{{\rm{n}}})$$

3 $${{\rm{E}}}_{{\rm{PM}},{\rm{y}}}=\sum _{{\rm{i}},{\rm{j}},{\rm{k}},{\rm{m}}}\sum _{{\rm{n}}}{{\rm{A}}}_{{\rm{i}},{\rm{j}},{\rm{k}},{\rm{m}}}\times {{\rm{AC}}}_{{\rm{i}},{\rm{m}}}\times (1-{{\rm{ar}}}_{{\rm{i}},{\rm{j}},{\rm{k}},{\rm{m}}})\times {{\rm{f}}}_{{\rm{k}},{\rm{y}}}\times {{\rm{X}}}_{{\rm{k}},{\rm{n}}}\times (1-{{\rm{\eta }}}_{{\rm{n}},{\rm{y}}})$$

4 $${{\rm{E}}}_{{\rm{P}}}=\sum _{{\rm{i}},{\rm{j}},{\rm{k}},{\rm{m}}}\sum _{{\rm{n}}}{{\rm{A}}}_{{\rm{i}},{\rm{j}},{\rm{k}},{\rm{m}}}\times {{\rm{X}}}_{{\rm{j}},{\rm{k}},{\rm{m}}}\times {{\rm{EF}}}_{{\rm{j}},{\rm{k}},{\rm{m}}}$$

where i represents the i^th^ plant; j represents the economic sector; k represents the fuel type; m represents the type of combustion; n represents emission control technology; y represents the particle size; A represents the activity rate; Scont is the sulfur content of fuels; Sr is the percentage of sulfur retained in the ash; AC is the ash content of the fuel; ar is the percentage of ashes as bottom ashes; f is the particulate mass fraction by size; X is the fraction of fuels or production for a sector; EF is the emission factor; and η_n_ is the removal efficiency of control technology n.

Data of air pollutants in Beijing were from the statistical yearbook^45^ while the emission data of major pollutants statistical for other cities in the Beijing-Tianjin-Hebei region were from MEIC emission inventory database (http://www.meicmodel.org/). MEIC is a complete emission inventory database, including the source classification and grading system, emission factor database, dynamic emission inventory methods, multi-scale high resolution emission models and a number of cloud computing platforms^46^.

**Figure 7:**
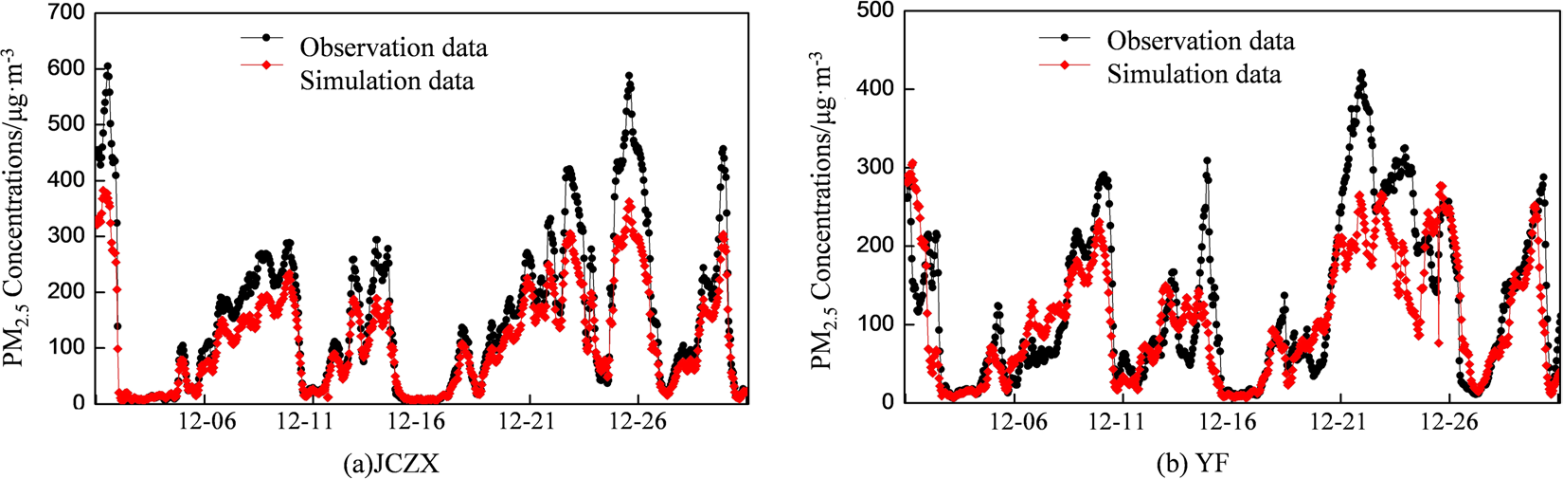
Comparisons between observed and simulated PM_2.5_ concentrations in December 2015 at two stations in Beijing.

Simulation results of the CAM*x* model were compared with the observation data (Fig. 7 **)**. The correlation coefficients of simulated and observed PM_2.5_ concentrations at two sites in Beijing were calculated with *R* between 0.69 and 0.80 and a general agreement was found between the simulation and observation data with more than 85% of data points falling into the siege area of 1:2 and 2:1 lines. Thus, the model simulation results provided solid references for the following analysis. Remaining deviations in the CAM*x* simulation results may be attributed to (1) insufficient chemical reaction mechanisms that fail to provide a comprehensive description of all atmospheric chemical reactions; (2) the uncertainty in the emission inventory, e.g., sea salt and dust particles are ignored in the emission inventories; and (3) meteorological field simulation errors. For instance, the thickness of boundary layer has an influence on the accuracy of simulation results^47, 48^.

### Analogy methods

Zhang *et al*.^16^ brought up an evaluation method for comparing concentrations of airborne pollutants under similar meteorological conditions and evaluating the net effectiveness of emergency measures. Generally, meteorological conditions for each air pollution event are of certain differences. For a proper evaluation, we averaged meteorological conditions in all heavily polluted days. Following this, we compared the ratio of PM_2.5_ to other pollutants during the two red alerts and that in other heavily polluted days in 2015. The life time of CO in the atmosphere ranges between 1 and 2 months, so concentrations of CO is generally stable, even in case of heavy pollution episodes. In addition to CO, the variations of SO_2_ concentrations are limited as well, owing to stable meteorological conditions. On the other hand, NO_2_. is an important indicator for vehicle emission. In this case, for this research, we compared the variations of PM_2.5_/SO_2_, PM_2.5_/CO, PM_2.5_/NO_2_ and NO_2_/CO, which were influenced by emission reduction measures, during the two alerts with the variations of these ratio values in other heavily polluted days in 2015.

## Electronic supplementary material

Supporting information
