# Supplementary material for: Spatio-temporal variations of PM2.5 concentrations and the evaluation of emission reduction measures during two red air pollution alerts in Beijing
Source: Sci Rep. 2017 Aug 15;7:8220. doi: 10.1038/s41598-017-08895-x (PMC5557902; doi:10.1038/s41598-017-08895-x)
Supplement: Supplementary file 1 — Supporting information [file 41598_2017_8895_MOESM1_ESM.doc]

**Spatio-temporal variations of PM_2.5_ concentrations and the evaluation of emission reduction measures during two red air pollution alerts in Beijing**

Nianliang Cheng^1,2,3^, Dawei Zhang^2,4^, Yunting Li ^2^, Xiaoming Xie^5,6^, Ziyue Chen^5*^, Fan Meng^1,3^, Bingbo Gao^6^, Bin He^5^

^1^ College of Water Sciences, Beijing Normal University, Beijing 100875,China

^2^ Beijing Municipal Environmental Monitoring Center, Beijing 100048, China

^3^Chinese Research Academy of Environmental Sciences, Beijing 100012, China

^4^Department of Environmental Science and Engineering, Tsinghua University, Beijing 100084, China

^5^ College of Global Change and Earth System Science, Beijing Normal University, 19 Xinjiekouwai Street, Haidian, Beijing, 100875, P. R. China

^6^  National Engineering Research Center for Information Technology in Agriculture, 11 Shuguang Huayuan Middle Road, Beijing 100097, China

^*^Corresponding author: Ziyue Chen, [zychen@bnu.edu.cn](mailto:zychen@bnu.edu.cn).

**Supporting Information**

**Supplementary Table 1 Specific requirements and emergency measures for the four air pollution alerts in Beijing**

| **Alert categrory** | **Alert level** | **Starting condition** | **Release**  **time** | **Release requirements** | **Emergency Measures** |
| --- | --- | --- | --- | --- | --- |
| Blue | Fourth | Heavy air pollution predicted to last for 24 hours | Real time | Heavy air pollution emergency headquarters office approves and organizes the alert release | Recommendated health protection measures,  Recommendation emergency measures |
| Yellow | Third | Heavy air pollution predicted to last for 48 hours | Early release | Heavy air pollution emergency headquarters office approves and organizes the alert release | Recommendated health protection measures  Recommendated emergency measures  Mandatory emergency measures(lockouts,flushing roads) |
| Orange | Second | Heavy air pollution predicted to last for 72 hours | 24 hours ahead | Heavy air pollution emergency headquarters commander approves and the office organizes the alert release | Recommendated health protection measures  Recommendated emergency measures,  Mandatory emergency measures( stopping the outdoor construction work, limiting production,banning fireworks and outdoor barbecue) |
| Red | First | Heavy air pollution predicted to last for more than 72 hours | 24 hours ahead | Emergency commission director approves and the emergency management office organizes the alert release | Recommendated health protection measures  Recommendated emergency measures  Mandatory emergency measures( stopping the outdoor construction work, limiting production,suspending the class, odd-even license plate policy,banning fireworks and outdoor barbecue) |

**Supplementary Table2. Simulated daily averaged PM_2.5_ emisssions in the Beijing-Tianjin-Hebei regions during two red alerts (unit: t)**

| PM2.5 emissions/t | | The first alert | | | | | | | | **The second alert** | | | | | | | |
| --- | --- | --- | --- | --- | --- | --- | --- | --- | --- | --- | --- | --- | --- | --- | --- | --- | --- |
|  |  | Dec 03 | Dec 04 | Dec 05 | Dec 06 | Dec 07 | Dec 08 | Dec 09 | Dec 10 | Dec 15 | Dec 16 | Dec 17 | Dec 18 | Dec 19 | Dec 20 | Dec 21 | Dec 22 |
| Beijing | 0 day |  |  |  |  | 148 | 135 | 135 | 135 |  |  |  |  | 135 | 135 | 135 | 135 |
|  | 1 day |  |  |  | 148 | 135 | 135 | 135 | 135 |  |  |  | 135 | 135 | 135 | 135 | 135 |
|  | 2 days |  |  | 148 | 135 | 135 | 135 | 135 | 135 |  |  | 135 | 135 | 135 | 135 | 135 | 135 |
|  | 3 days |  | 148 | 135 | 135 | 135 | 135 | 135 | 135 |  | 135 | 135 | 135 | 135 | 135 | 135 | 135 |
|  | 4 days | 148 | 135 | 135 | 135 | 135 | 135 | 135 | 135 | 135 | 135 | 135 | 135 | 135 | 135 | 135 | 135 |
| Tianjin  * | 0 day |  |  |  |  | 257 | 237 | 237 | 237 |  |  |  |  | 237 | 237 | 237 | 237 |
|  | 1 day |  |  |  | 257 | 237 | 237 | 237 | 237 |  |  |  | 237 | 237 | 237 | 237 | 237 |
|  | 2 days |  |  | 257 | 237 | 237 | 237 | 237 | 237 |  |  | 237 | 237 | 237 | 237 | 237 | 237 |
|  | 3 days |  | 257 | 237 | 237 | 237 | 237 | 237 | 237 |  | 237 | 237 | 237 | 237 | 237 | 237 | 237 |
|  | 4 days | 257 | 237 | 237 | 237 | 237 | 237 | 237 | 237 | 237 | 237 | 237 | 237 | 237 | 237 | 237 | 237 |
| Hebei  * | 0 day |  |  |  |  | 2091 | 1890 | 1890 | 1890 |  |  |  |  | 1890 | 1890 | 1890 | 1890 |
|  | 1 day |  |  |  | 2091 | 1890 | 1890 | 1890 | 1890 |  |  |  | 1890 | 1890 | 1890 | 1890 | 1890 |
|  | 2 days |  |  | 2091 | 1890 | 1890 | 1890 | 1890 | 1890 |  |  | 1890 | 1890 | 1890 | 1890 | 1890 | 1890 |
|  | 3 days |  | 2091 | 1890 | 1890 | 1890 | 1890 | 1890 | 1890 |  | 1890 | 1890 | 1890 | 1890 | 1890 | 1890 | 1890 |
|  | 4 days | 2091 | 1890 | 1890 | 1890 | 1890 | 1890 | 1890 | 1890 | 1890 | 1890 | 1890 | 1890 | 1890 | 1890 | 1890 | 1890 |

**(***We assumed that the air pollution alert level in Tianjin and Hebei was the same to that in Beijing)
